# Decoding Neuromuscular Disorders: The Complex Role of Genetic and Epigenetic Regulators

**DOI:** 10.3390/genes16060622

**Published:** 2025-05-23

**Authors:** Bladimir Roque-Ramírez, Karla Estefanía Ríos-López, Luz Berenice López-Hernández

**Affiliations:** 1 Laboratorio de Nutrigenética y Nutrigenómica, Instituto Nacional de Medicina Genómica, Periférico Sur 4809, Mexico City 14610, Mexico; broque@inmegen.gob.mx (B.R.-R.);; 2 Universidad Autónoma de Occidente, Los Mochis 81216, Mexico; 3 Departamento Académico de Ciclo de Vida, Universidad Autónoma de Guadalajara, Av Patria 1201, Zapopan 45129, Mexico

**Keywords:** neuromuscular disorders, epigenetics, nutrients, ALS, SMA, muscular dystrophies

## Abstract

Neuromuscular disorders (NMDs), such as amyotrophic lateral sclerosis (ALS), spinal muscular atrophy (SMA), and muscular dystrophies (e.g., Duchenne muscular dystrophy, DMD), are primarily driven by genetic mutations but are critically modulated by epigenetic mechanisms such as DNA methylation, histone modifications, and noncoding RNA activity. These epigenetic processes contribute to phenotypic variability and disease progression, and emerging evidence suggests that environmental factors, particularly nutrition and exercise, may further influence the molecular pathways that modulate these diseases. Dietary bioactive compounds (e.g., polyphenols and omega-3 fatty acids) exhibit epigenetic modulatory properties, which could mitigate oxidative stress, inflammation, and muscle degeneration in NMDs. For example, the inhibition of DNMTs and HDACs by curcumin in ALS models and the promyogenic effects of green tea catechins in DMD suggest plausible, though still requiring investigation, therapeutic avenues. However, the clinical application of nutriepigenetic interventions is preliminary and requires further validation. This review examines the interaction of genetic and epigenetic factors in ALS, SMA, and muscular dystrophies, highlighting their combined role in the heterogeneity of these diseases. Integrative therapeutic strategies combining gene therapies, epigenetic modulators, and lifestyle interventions may offer a multidimensional approach to the management of NMD. A deeper understanding of these interactions will be essential for advancing precision medicine and improving patient outcomes.

## 1. Introduction

Neuromuscular disorders (NMDs) represent a clinically and genetically heterogeneous group of diseases, primarily of genetic origin, though acquired forms (e.g., autoimmune myasthenia gravis or toxic neuropathies) also exist [1]. These disorders disrupt the function of motor neurons, peripheral nerves, neuromuscular junctions, or skeletal muscle, leading to progressive weakness, sensory deficits (in neuropathies), and/or impaired nerve-to-muscle signaling [2].

NMDs are classified into the following major subtypes: muscular dystrophies (MDs) such as Duchenne muscular dystrophy (DMD) and facioscapulohumeral muscular dystrophy (FSHD); motor neuron diseases such as amyotrophic lateral sclerosis (ALS) and spinal muscular atrophy (SMA); peripheral neuropathies (e.g., Charcot–Marie–Tooth disease [CMT]); neuromuscular junction disorders (e.g., autoimmune myasthenia gravis [MG]); myopathies (congenital, metabolic, or myotonic); and ion channelopathies (e.g., periodic paralysis) [3,4].

While their etiology varies, shared pathological features include muscle wasting, contractures, and, in some cases, cardiomyopathy or respiratory insufficiency [2]. ALS, the most common motor neuron disease, and SMA, a monogenic lower motor neuron disorder, illustrate the spectrum of genetic mechanisms [3]. Similarly, MG caused by autoantibodies targeting acetylcholine receptors exemplifies synaptic dysfunction in NMDs [4].

In NMDs, the age of onset, severity, and progression differ significantly even within the same disease, reflecting the influence of genetic modifiers (e.g., *SMN2* copy number in SMA) and environmental factors [1,3]. NMDs arise primarily from genetic variants, including intragenic mutations, copy number variations (deletions/duplications), and single-nucleotide changes, which disrupt proteins critical for neuromuscular function [5]. However, disease pathogenesis can also stem from any mechanism impairing the production or activity of these proteins, such as aberrant RNA processing or post-translational modifications [6].

Epigenetic regulation further modulates NMD phenotypes through heritable, non-sequence-based modifications of chromatin structure. These mechanisms—DNA methylation, histone post-translational modifications (e.g., acetylation, methylation), and non-coding RNAs (ncRNAs)—dynamically control gene expression by altering chromatin accessibility [6]. For instance, DNA hypermethylation may silence protective genes in muscular dystrophies, while ncRNAs can fine-tune disease-related pathways in motor neuron diseases [7,8].

Due to these layered regulatory influences, genotype-phenotype correlations in NMDs are often complex. Phenotypic variability reflects not only the primary genetic variant but also modifier genes, epigenetic states, and environmental interactions [5]. Herein, we discuss the most relevant genetic and epigenetic factors involved in NMDs, drawing on evidence from both human studies and animal models, with the aim to provide a deeper understanding of the complex interplay between genetic and epigenetic factors in shaping the clinical manifestations of NMDs.

## 2. Genetic Mechanisms in Neuromuscular Disorders

### Disease Heterogeneity and Genetic Modifiers

Establishing clear genotype–phenotype correlations in NMDs remains challenging due to variable expressivity, incomplete penetrance, and additional mechanisms. For instance, Schwartz et al. (2007) documented an asymptomatic male with a dystrophin exon 16 deletion that is typically pathogenic (that would be expected to cause DMD/BMD) who displayed normal muscle strength and histology [9]. Conversely, Chiba et al. (2003) reported two sisters sharing the same dysferlin pathogenic variant but exhibiting divergent phenotypes: one with Miyoshi myopathy and the other with limb-girdle muscular dystrophy (LGMD) [10]. Such discordances highlight the influence of genetic modifiers and epigenetic factors on disease phenotypes.

Genetic modifiers are genes that alter the phenotypic impact of a primary pathogenic variant without directly causing disease [3,11]. They modulate disease severity, age of onset, or progression by affecting gene expression, protein function, or compensatory pathways [12,13]. Notably, this mechanism differs from oligogenic inheritance, where pathogenic variants in multiple genes are required for disease manifestation. For example, FSHD2 requires both a pathogenic *SMCHD1* variant and a permissive D4Z4 allele [3]. The genetic landscape of NMDs extends beyond simple biallelic loss-of-function mutations, encompassing modifier variants, polygenic contributions, and complex allelic interactions that collectively shape disease phenotypes. While autosomal recessive forms classically require biallelic pathogenic variants, the frequent involvement of multiple genes, variant combinations, and modifier alleles creates substantial diagnostic challenges [14]. This genetic complexity underscores the critical need for curated gene-specific databases that integrate genotype-phenotype correlations, functional validation data, and epigenetic annotations. Such resources are indispensable for resolving variant interpretation ambiguities, elucidating polygenic contributions, and advancing precision medicine in NMDs to ultimately bridge the gap between molecular diagnostics and clinical outcomes [15,16,17].

## 3. Epigenetic Regulation in Neuromuscular Disorders

Epigenetic mechanisms, including DNA methylation, histone modifications, and non-coding RNAs (ncRNAs), alongside genetic modifiers, critically influence NMD phenotypes by modulating gene expression and cellular functions. Consequently, clinical manifestations reflect not only the primary pathogenic variant but also the interplay of secondary genetic and environmental factors, contributing to phenotypic diversity and potential therapeutic avenues.

### 3.1. DNA Methylation in NMDs

**Figure 1:**
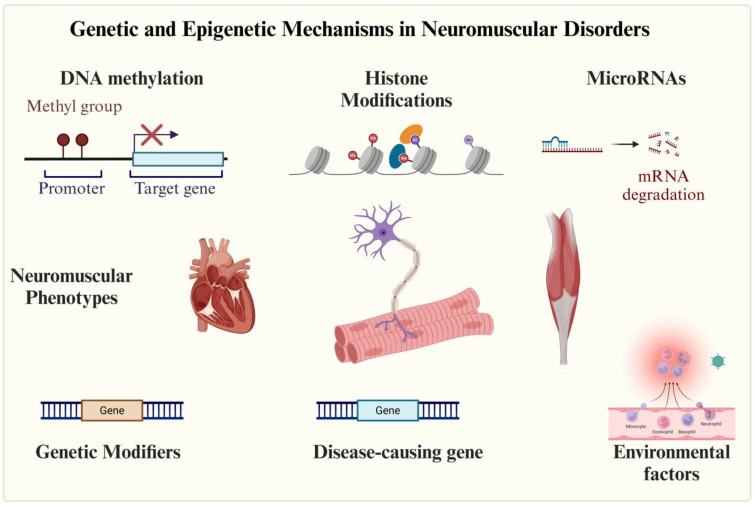
Epigenetic mechanisms involved in neuromuscular disorders. Neuromuscular diseases are primarily of genetic origin, though environmental factors may also contribute. Epigenetic mechanisms play a role in both disease development and phenotypic modulation.

DNA methylation, mediated by DNA methyltransferases (DNMTs), entails the addition of a methyl group to cytosine residues primarily at CpG dinucleotides (Figure 1). This modification exhibits context-dependent effects: (1) promoter methylation typically represses gene expression, and (2) gene body methylation suppresses spurious transcription and is associated with actively transcribed genes [18].

While not universally observed in NMDs, DNA methylation alterations are well-documented in specific disorders. For example, in MD1, hypermethylation of the expanded *DMPK* CTG repeat region correlates with disease severity and somatic instability [8,19]. Similar epigenetic dysregulation has been implicated in FSHD, where hypomethylation of the *D4Z4* repeat is pathogenic [5] Interestingly, recent findings demonstrated that DNA methylation levels at the *D4Z4* macrosatellite correlate with clinical severity in FSHD, independent of repeat size. These epigenetic markers show a stronger association with disease manifestations than genetic parameters alone, suggesting DNA methylation status may serve as a more accurate biomarker for monitoring FSHD progression [20].

### 3.2. Histone Modifications and Chromatin Remodeling in NMDs

Histone post-translational modifications (PTMs) including acetylation, methylation, phosphorylation, ubiquitylation, and sumoylation dynamically regulate chromatin structure and gene expression by modulating transitions between transcriptionally active (euchromatin) and repressive (heterochromatin) states. These processes are increasingly implicated in NMD pathogenesis and therapy. While not all NMDs involve primary histone alterations, emerging evidence underscores their role in disease progression. For example, in DMD, the histone deacetylase inhibitor (HDACi) givinostat reduces fibrosis and improves mitochondrial function by promoting histone acetylation, thereby reactivating compensatory mechanisms such as utrophin expression [21]. Givinostat is under clinical evaluation for non-ambulant DMD (NCT05933057) and was previously trialed in BMD (BMD; NCT03238235).

In SMA, HDACis (e.g., AR42, 4PBA, TSA) reverse repressive marks (e.g., H3K9/27 deacetylation) at the *SMN2* locus, enhancing exon 7 inclusion and SMN protein levels. Neuronal iPSC models from SMA patients demonstrated that HDACis not only upregulate *SMN2* expression but also restore SMN nuclear gems [22]. These findings suggest broader applicability for HDACis in NMDs with transcriptional dysregulation, such as FSHD (linked to *D4Z4* chromatin relaxation) and DM1 (associated with *DMPK* locus silencing). By reactivating compensatory genes and mitigating degeneration, epigenetic therapies represent a promising strategy for NMD treatment (Figure 1) [19,23].

### 3.3. Non-Coding RNAs in NMDs

Non-coding RNAs (ncRNAs), including microRNAs (miRNAs) and long non-coding RNAs (lncRNAs), serve as crucial epigenetic regulators in neuromuscular disorders (NMDs). MyomiRs, a muscle-specific miRNA subclass (e.g., miR-1, miR-133a/b, miR-206, miR-208a/b, miR-486, and miR-499), are particularly important for skeletal muscle development, maintenance, and regeneration [24]. These miRNAs function by binding to the 3′ untranslated region (3′UTR) of target mRNAs to post-transcriptionally silence gene expression through mRNA degradation or translational repression. Specific examples include miR-1 promoting differentiation via HDAC4 and IGF-1 targeting, miR-133a/b enhancing myoblast fusion and reducing fibrosis through *SRF* and *NELF-A* modulation, and miR-206 activating satellite cells by inhibiting Pax7 [25]. Dysregulation of these miRNAs contributes to NMD pathology, as seen in DMD where miR-486 downregulation impairs muscle regeneration [26]. Current therapeutic investigations focus on miRNA modulation, including miR-29 antagonists for fibrosis reduction, miR-206 mimics to boost regeneration, and miR-208a inhibitors for cardiomyopathy management [27]. Together, these findings underscore the potential of ncRNA-based therapies to target NMDs at the epigenetic level (Figure 1).

## 4. Disease-Specific Genetic and Epigenetic Mechanisms

### 4.1. Amyotrophic Lateral Sclerosis

**Figure 2:**
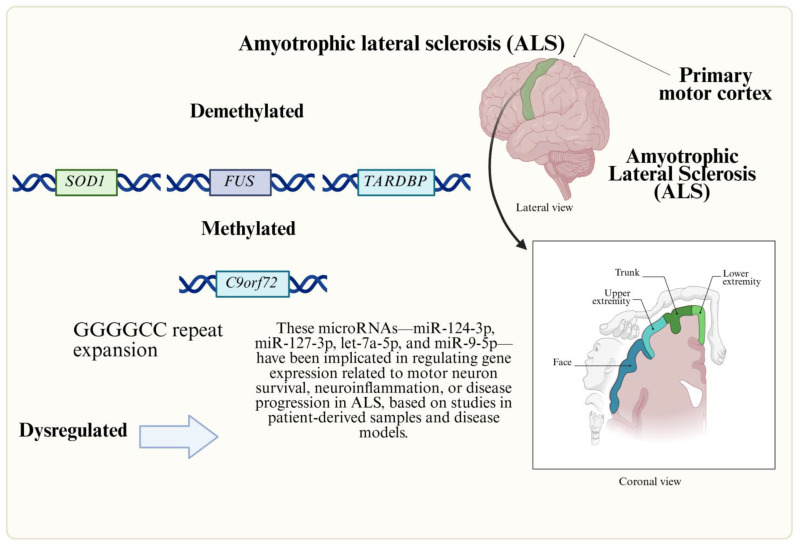
Genetic and epigenetic factors involved in ALS. ALS is an NMD linked to motor neuron degeneration. Altered RNA, histone acetylation, and miRNA pathways drive pathology, impacting cognitive function, survival, and therapeutic targets.

ALS is a fatal neurodegenerative disorder characterized by the progressive degeneration of the upper and lower motor neurons, leading to muscle weakness, paralysis, and, in 30–50% of cases, frontotemporal dementia (FTD)-like cognitive deficits. Approximately 90% of ALS cases are sporadic, while 5–10% are familial, with pathogenic variants in *SOD1*, *TARDBP*, *FUS*, and *C9orf72* (GGGGCC hexanucleotide repeat expansions) accounting for over 50% of inherited forms (Figure 2). The disease pathogenesis involves multiple mechanisms, including protein misfolding (e.g., TDP-43 aggregates), dysregulated RNA metabolism, cytoskeletal dysfunction, and neuroinflammation (Table 1) [28]. Genetic modifiers and epigenetic alterations significantly influence disease onset, progression, and phenotypic heterogeneity. Key examples include the following:

- The *UNC13A* rs12608932 variant, where the C allele shortens survival by 5–12 months, delays onset, and increases ALS-FTD comorbidity, likely by promoting nonsense-mediated decay of *UNC13A* transcripts and exacerbating TDP-43 aggregation.
- Intermediate-length *ATXN2* polyglutamine repeats (27–33), which elevate ALS risk; inhibition of *ATXN2* reduces TDP-43 pathology and improves motor function in models [29].
- The *FRMD8/NEAT1* locus variant (rs10128627), associated with earlier onset in Chinese cohorts, possibly through increased expression of the long non-coding RNA NEAT1 [27].

Epigenetic dysregulation, particularly DNA methylation, plays a critical role in ALS. Genome-wide association studies have linked methylation changes to disease variability, including hypermethylation of the *C9orf72* promoter in patients with GGGGCC expansions, which suppresses gene expression, and hypomethylation of *SOD1*, *FUS*, and *TARDBP*, potentially increasing their expression [30]. Twin studies reveal accelerated epigenetic aging in ALS patients, with advanced methylation age compared to controls [31]. Additionally, methylation alterations disrupt pathways involved in calcium homeostasis, neurotransmission, oxidative stress, and neuroinflammation in ALS-affected tissues [30]. Aberrant methylation in the mitochondrial D-loop region further implicates epigenetic dysregulation in mitochondrial dysfunction [32].

In amyotrophic lateral sclerosis (ALS), the dysregulation of histone acetylation contributes to impaired axonal transport, a key pathological mechanism. Reduced histone acetylation, associated with diminished histone acetyltransferase (HAT) activity, has been observed in ALS models [33]. Conversely, HDACs exhibit dual roles, with some isoforms promoting neurodegeneration, while others may confer neuroprotection [34]. Although HDACis show therapeutic promise in preclinical models, their efficacy remains partial, failing to halt all disease manifestations. Additionally, aberrant histone methylation and heterochromatin reorganization are implicated in *C9orf72*-related ALS, suggesting a broader epigenetic disruption in the disease [35,36].

Beyond histone modifications, altered microRNA (miRNA) expression influences ALS pathogenesis by disrupting neurogenesis, synaptic integrity, and neuroinflammation. ALS-associated proteins, including TDP-43, FUS, and SOD1, share functional roles in miRNA processing, and their mutations or aggregations can dysregulate miRNA pathways [37]. *C9orf72* hexanucleotide repeat expansions exacerbate this dysregulation by sequestering RNA-binding proteins, further impairing RNA metabolism. Specific miRNAs, such as miR-9-5p, are consistently dysregulated across neurodegenerative diseases [27]. Postmortem studies of sporadic ALS spinal cord tissues reveal elevated miR-155 and miR-142-5p alongside reductions in let-7e, miR-148b-5p, and miR-133b, though further validation is needed to confirm their diagnostic or mechanistic relevance [38].

Large-scale epigenome-wide association studies (EWASs) have identified 45 differentially methylated positions (DMPs) linked to 42 genes in over 9000 ALS cases, with enrichment in metabolic, cholesterol biosynthesis, and immune pathways [39]. Methylation analyses associate high-density lipoprotein (HDL) cholesterol, body mass index (BMI), leukocyte ratios, and alcohol consumption with ALS risk. Integration with genome-wide association study (GWAS) data further implicates cholesterol biosynthesis in disease pathogenesis. Notably, specific DMPs and blood cell composition estimates correlate with survival, highlighting their potential as progression biomarkers and therapeutic targets.

**Table 1:** Key genetic and epigenetic modifiers in ALS.

Category	Key Factor	Effect on ALS	Mechanism	References
Genetic Modifiers	*UNC13A* (rs12608932-C)	Decreases survival by 5–12 months; increases risk of bulbar onset and frontotemporal dementia (FTD)	Promotes aggregation of hyperphosphorylated TDP-43; enhances decay of *UNC13A* transcript via NMD	[29]
	*ATXN2* (intermediate polyQ)	Increases ALS risk (non-causal relationship)	Modulates TDP-43 aggregation; potential therapeutic target	[29]
	*FRMD8* (rs10128627)	Associated with earlier onset in Chinese cohorts	Increases NEAT1 lncRNA expression	[40]
Epigenetic Factors	Global DNA methylation	Correlates with disease severity (increased in SOD1 carriers)	Affects calcium homeostasis and neuroinflammation	[30,41]
	*C9orf72* promoter methylation	Silences gene expression in *C9orf72* repeat carriers	Alters RNA metabolism and facilitates protein sequestration	[32,42]
	Mitochondrial DNA (D-loop)	Contributes to mitochondrial dysfunction	Associated with altered methylation patterns	[32]
	HDAC dysregulation	Impairs axonal transport	Decreases histone acetylation; effects of mixed HDAC inhibitors	[33,34]
	MicroRNA dysregulation	Alters neuroinflammation and survival	Increased levels of miR-155 and decreased levels of let-7e and miR-133b, among others	[38]
	45 Differentially Methylated Positions across 42 genes	Links to cholesterol metabolism, immunity, and survival	Potential biomarkers for disease progression and therapy	[39]

### 4.2. Spinal Muscular Atrophy

**Figure 3:**
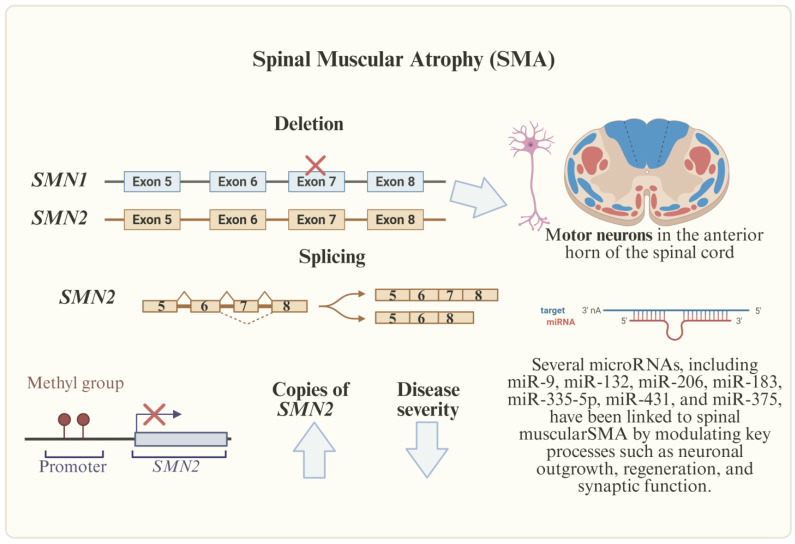
Genetic and epigenetic factors involved in SMA. SMA, caused by *SMN1* deletions, is influenced by *SMN2* copies, methylation, and deregulated miRNAs (e.g., miR-1-3p, miR-133b), which hold promise as biomarkers and therapeutic targets.

Spinal muscular atrophy (SMA) is an autosomal recessive disorder characterized by the degeneration of α motor neurons in the spinal cord, resulting in progressive muscle weakness and atrophy (Figure 3). The disease is classified into five subtypes (0–IV) based on the age of onset and motor function. Type 0, the most severe form, presents prenatally with profound weakness and respiratory failure at birth, often leading to perinatal death. Type I (infantile-onset, ~60% of cases) manifests before six months with hypotonia, inability to sit independently, and respiratory compromise, historically resulting in mortality by age two without intervention. Type II (intermediate) emerges between 6 and 18 months, permitting sitting but not independent ambulation, with survival into adulthood (30–50 years). Type III (juvenile-onset) presents after 18 months, initially allowing walking but often leading to the progressive loss of ambulation, while Type IV (adult-onset) exhibits mild motor impairment around 18–21 years and near-normal lifespan [4,43,44].

At the molecular level, SMA is caused by homozygous deletions or loss-of-function mutations in *SMN1*, which encodes the survival motor neuron (SMN) protein essential for motor neuron viability. While *SMN1* produces full-length functional SMN protein, its paralog *SMN2* predominantly generates truncated, non-functional isoforms due to a critical C-to-T transition in exon 7 that leads to aberrant splicing and exon skipping. This molecular defect explains why *SMN1* deficiency causes SMA, while *SMN2* copy number (typically 1–5 copies in humans) serves as the primary phenotypic modifier, with higher copy numbers generally correlating with milder disease manifestations. The inverse relationship between *SMN2* copy number and disease severity has important therapeutic implications, as strategies to modulate *SMN2* splicing can potentially compensate for *SMN1* deficiency [43]. Recent research has identified specific intronic splicing silencers (notably ISS-N1) in *SMN2* that repress exon 7 inclusion, and this discovery led to the development of nusinersen (Spinraza^®^), an antisense oligonucleotide therapy that binds ISS-N1 to promote exon 7 inclusion and increase the production of functional SMN protein [45]. Beyond splicing regulation, emerging evidence highlights the role of epigenetic mechanisms in SMA pathogenesis, including DNA methylation patterns of *SMN2* and other neuronal genes (e.g., *PAX6*, *CHAT*) that influence disease variability. Studies using SMA patient-derived induced pluripotent stem cells (iPSCs) have revealed dysregulated methylation profiles during motor neuron differentiation, suggesting that epigenetic modifications may contribute to the progressive nature of the motor neuron degeneration in SMA [4,43,46].

In addition, abnormal miRNA expression is linked to SMA; therefore, miRNAs have the potential to serve as biomarkers of disease progression, therapy response, and also for understanding different clinical outcomes. The most frequently deregulated miRNAs in SMA patients include miR-1-3p, miR-133a-3p, miR-133b, and miR-206 (Table 2). These miRNAs offer promising possibilities for improving patient classification and monitoring disease progression and response to treatments [47].

### 4.3. Muscular Dystrophies

Muscular dystrophies (MDs) are a genetically and clinically heterogeneous group of neuromuscular disorders (NMDs) characterized by progressive skeletal muscle degeneration, fibrosis, and functional impairment. Although they share common pathological features—such as muscle fiber necrosis, chronic inflammation, and fibrofatty replacement—their etiologies, clinical progression, and systemic manifestations vary significantly (Table 3) [48].

#### 4.3.1. Myotonic Dystrophy

MD1 and MD2 are autosomal dominant disorders caused by non-coding repeat expansions in *DMPK* (CTG) and *CNBP* (CCTG), respectively [49]. DM1 exhibits severe congenital and childhood-onset forms, distal myotonia, and profound systemic involvement, including cardiac arrhythmias, cataracts, and endocrine dysfunction. In contrast, DM2 typically presents in adulthood with proximal weakness, myalgia, and milder systemic features. Cognitive impairment and excessive daytime sleepiness are more prevalent in DM1 than DM2. Genetic anticipation, due to repeat instability, is well-documented in DM1 but remains unclear in DM2 [49,50,51].

#### 4.3.2. Duchenne and Becker Muscular Dystrophy

**Figure 4:**
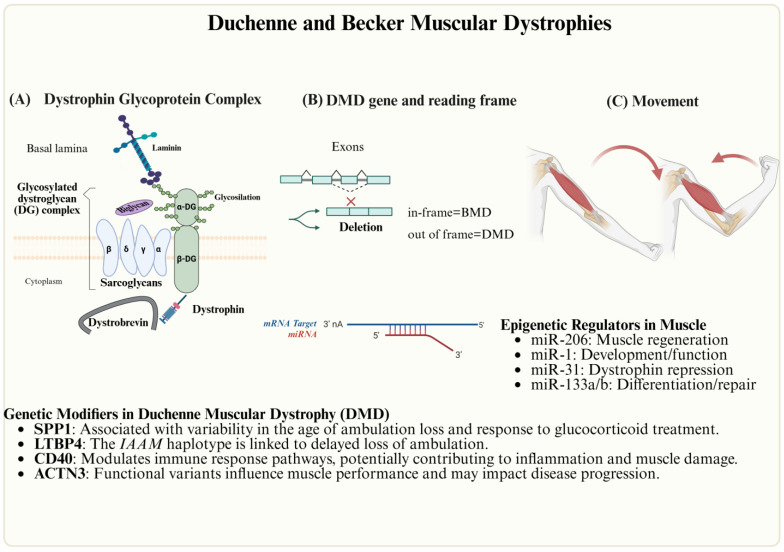
Genetic and epigenetic factors involved in DMD. (**A**) Dystrophin Glycoprotein Complex, (**B**) the *DMD* gene, and (**C**) muscular contraction.

DMD, the most common childhood MD, results from loss-of-function mutations in the dystrophin gene (Xp21.2), leading to near-complete dystrophin deficiency (typically due to out-of-frame deletions) (Figure 4). DMD manifests as progressive weakness, loss of ambulation by adolescence, and reduced life expectancy (median survival ~30 years with optimal care). BMD, caused by in-frame deletions producing partially functional dystrophin, presents with milder, later-onset weakness and slower progression, often with survival beyond middle age. Both disorders involve cardiac and respiratory complications necessitating multidisciplinary management [48,52].

#### 4.3.3. Limb-Girdle Muscular Dystrophies

LGMDs encompass over 30 subtypes with autosomal dominant (LGMD1) or recessive (LGMD2) inheritance, characterized by progressive proximal weakness affecting the pelvic and shoulder girdles. Causative mutations disrupt proteins critical for sarcolemmal integrity (e.g., dysferlin, sarcoglycans), sarcomeric function (e.g., titin), or extracellular matrix stability (e.g., collagen VI). Phenotypic variability includes distal involvement or cardiac/respiratory complications in severe subtypes. Disease progression ranges from mild ambulatory impairment to loss of independent mobility. Unlike myotonic dystrophies, LGMDs lack systemic myotonia or pronounced multisystem involvement [53,54].

#### 4.3.4. Facioscapulohumeral Dystrophy

FSHD is caused by pathogenic D4Z4 repeat contractions (1–10 units) on a permissive 4qA haplotype (FSHD1) or mutations in chromatin-modifying genes (*SMCHD1*, *DNMT3B*, *LRIF1*) with a 4qA allele (FSHD2) [55]. Both forms lead to aberrant *DUX4* expression, driving muscle degeneration. Clinical severity varies from mild facial and scapular weakness to severe respiratory compromise, influenced by the repeat size, somatic mosaicism, and epigenetic dysregulation (e.g., 4q35 hypomethylation) [55,56,57].

In DMD, dysregulated non-coding RNAs (e.g., miR-1, miR-206, miR-29) and HDACs impair muscle regeneration by suppressing myogenic transcription factors. HDAC inhibitors (e.g., givinostat) show promise in restoring epigenetic homeostasis and improving muscle function in preclinical and clinical trials, though their efficacy depends on the disease stage and individual variability [21].

Epigenetic regulation also governs vascular smooth muscle cell (SMC) plasticity. H3K4me2 demethylation at SMC-specific promoters disrupts lineage identity, reducing contractility and TET2 recruitment. Concomitant miR-145 suppression exacerbates phenotypic switching, implicating the H3K4me2-TET2-miR-145 axis in vascular pathologies [58].

**Table 3:** Key genetic and epigenetic modifiers in muscular dystrophies.

Category	Key Factors	Mechanism/Function	Implications	References
Genetic Modifiers	*DMPK* (DM1), *CNBP* (DM2)	CTG/CCTG repeat expansions lead to RNA toxicity and splicing dysregulation	Myotonia and multisystem involvement	[49]
	Dystrophin (DMD/BMD)	Disruption of sarcolemmal stability results in cytoskeletal detachment	Progressive muscle weakness and cardiomyopathy	[4]
	*DYSF*, *SGCA-SGCD*, *DMD*	Defects in the sarcolemmal/sarcoglycan complex cause membrane instability	Proximal muscle weakness with variable cardiac involvement	[48]
Epigenetic Regulators	*MEF2B*, *CBX3*, *SMC3*	Chromatin compaction leads to impaired muscle stem cell regeneration	Reduced muscle repair in DMD; reversible through MEF2B re-expression	[59]
	miR-1, miR-206, miR-29	Regulation of myogenesis is mediated by targeting HDAC4 and YY1	Dysregulation exacerbates muscle wasting	[59]
	lncMyoD, lincMD1	Chromatin remodeling and miRNA sequestration modulate muscle differentiation	Influence muscle differentiation processes	[59]
	HDACs (e.g., HDAC2/9)	Histone and non-histone deacetylation lead to transcriptional repression	Overexpression in dystrophic muscle; HDAC inhibitors enhance function	[59]
	H3K4me2-TET2-miR-145 axis	Maintains satellite muscle cell identity; demethylation causes loss of contractility	Vascular dysfunction in musculoskeletal disorders	[58]

## 5. Environmental and Lifestyle Modulators of NMDs

### 5.1. Exercise and Epigenetic Regulation in Muscles

Physical activity induces significant adaptations in skeletal muscle, contributing to improved metabolic health and a reduced risk of chronic diseases. These adaptations are driven by transcriptional reprogramming mediated by endurance or resistance training, which modulates metabolic and myogenic pathways. Epigenetic mechanisms including DNA methylation, histone modifications, and microRNAs (miRNAs) play a central role in regulating exercise-induced muscle plasticity by integrating environmental stimuli with gene expression [60,61].

The dynamic nature of epigenetic modifications is exemplified by rapid, exercise-induced changes in DNA methylation. In a study of 14 sedentary individuals, acute exercise triggered global hypomethylation in the promoters of genes associated with muscle plasticity (e.g., *PGC-1α*, *TFAM*, *MEF2A*, and *PDK4*), correlating with increased mRNA expression [62]. This effect was more pronounced following high-intensity exercise, demonstrating the responsiveness of skeletal muscle to metabolic demand.

Further insights into the persistence of exercise-induced epigenetic changes come from a study using an inducible myonuclear labeling system (HSA-GFP mice). Following an 8-week progressive weighted wheel running (PoWeR) regimen, myonuclei and interstitial nuclei exhibited distinct methylation patterns: myonuclei showed hypomethylation in promoters linked to Wnt signaling, while interstitial nuclei displayed hypermethylation in the same pathways [63]). After 12 weeks of detraining, myonuclei retained differential methylation at muscle remodeling-associated gene promoters, suggesting an epigenetic memory of prior training. Remarkably, retrained mice exhibited enhanced muscle growth without additional myonuclear accretion, implying that retained epigenetic modifications prime skeletal muscle for faster adaptation upon retraining.

These findings underscore the critical role of epigenetic regulation in exercise-induced muscle plasticity, with implications for optimizing training strategies and rehabilitation.

### 5.2. Nutriepigenetic Modulators in Neuromuscular Disorders

Emerging evidence indicates that dietary nutrients can modulate epigenetic regulation in NMDs through their influence on key epigenetic enzymes, including DNMTs, histone acetyltransferases (HATs), and HDACs [64]. Preclinical studies in animal models of NMDs demonstrate that targeted dietary interventions can ameliorate disease progression by modifying these epigenetic pathways (Table 4) [64].

Recent nutriepigenomic research has revealed that bioactive food components exert multi-level regulatory effects, influencing the following [65]:

- Chromatin organization and DNA accessibility,
- Transcriptional and translational processes,
- Post-translational modifications of histones.

Notably, specific natural compounds have shown potential to modify disease phenotypes in certain NMDs through epigenetic-mediated changes in gene expression [65]. These findings highlight the therapeutic potential of nutriepigenetic approaches, though further clinical validation is needed to establish optimal nutritional strategies for human applications.

### 5.3. Nutrients and Amyotrophic Lateral Sclerosis

As mentioned before, ALS is a progressive neurodegenerative disease characterized by motor neuron loss, driven by multiple pathological mechanisms, including oxidative stress, mitochondrial dysfunction, protein aggregation, excitotoxicity, neuroinflammation, and impaired axonal transport [66]. Epigenetic alterations, such as DNA hypermethylation and chromatin remodeling, are also implicated, with increased 5-methylcytosine (5 mC) levels and the elevated expression of DNA methyltransferases (DNMT1 and DNMT3a) observed in ALS motor neurons [67].

Curcumin, a polyphenol derived from turmeric, exhibits antioxidant, anti-inflammatory, and metal-chelating properties, making it a candidate for ALS therapy. Preclinical studies demonstrate that curcumin enhances the clearance of misfolded superoxide dismutase 1 (SOD1) by immune cells, reduces neuroinflammation, and inhibits amyloidogenic protein aggregation [68]. Molecular docking studies suggest curcumin may bind to the catalytic sites of DNMTs and HDACs, potentially reducing DNA methylation and increasing histone H4 acetylation, which could upregulate cytoprotective genes such as suppressors of cytokine signaling (SOCS1 and SOCS3) [69]. However, in vivo evidence linking these effects to motor neuron survival in ALS is limited. A small clinical trial (*n* = 50) found that nanocurcumin, administered for 12 months alongside standard ALS treatment, was well tolerated and associated with improved survival compared to controls [70]. These findings require validation in larger, randomized controlled trials due to curcumin’s poor bioavailability and the study’s small sample size.

Dietary restriction, including caloric restriction or intermittent fasting, may also modulate ALS pathology by inducing autophagy, a cellular process that clears protein aggregates and damaged organelles [71]. In ALS, autophagy facilitates the removal of toxic SOD1 and TAR DNA-binding protein 43 (TDP-43) aggregates, which are ubiquitinated, phosphorylated, and mislocalized to the cytoplasm [72]. Lipid peroxidation products, such as 4-hydroxy-2-nonenal (HNE), exacerbate TDP-43 pathology by promoting its insolubilization [73]. Autophagy induction has been shown to reduce neuronal apoptosis and slow disease progression in preclinical models [74]. However, excessive autophagy may accelerate neuronal loss in certain contexts, the mechanisms by which dietary restriction exerts benefits in ALS remain speculative, with limited clinical evidence supporting its efficacy. Therefore, curcumin and dietary restriction show promise in targeting oxidative stress, protein aggregation, neuroinflammation, and epigenetic dysregulation in ALS. While preclinical and early clinical data are encouraging, their therapeutic potential is constrained by the limited bioavailability, small-scale studies, and incomplete mechanistic understanding. Future research should prioritize large-scale clinical trials and mechanistic studies to validate these interventions and optimize their application in ALS management.

### 5.4. Nutrients and Spinal Muscular Atrophy

Spinal muscular atrophy (SMA) is a genetic neuromuscular disorder caused by mutations in the survival motor neuron 1 (SMN1) gene, leading to motor neuron degeneration, muscle weakness, and metabolic complications. While gene-modulating therapies, such as nusinersen and onasemnogene abeparvovec, have significantly improved survival and motor function [24], emerging evidence suggests that personalized dietary interventions, guided by nutrigenomics, could further optimize SMA outcomes by addressing neurodegeneration and metabolic comorbidities. Nutrigenomics examines how nutrients interact with genetic and molecular pathways, offering the potential to tailor diets to individual genetic profiles [75].

Bioactive compounds in certain foods show promise in preclinical SMA models. For instance, kiwifruit (Actinidia deliciosa), rich in antioxidants and anti-inflammatory compounds, improved motor neuron survival and locomotion in a *Caenorhabditis elegans* SMA model [76]. These effects may stem from reduced oxidative stress and inflammation, though the specific bioactive components remain unidentified. Similarly, flavonoids from green tea extract (GTE) and cocoa have been shown to enhance excitatory synaptic stability in motor neurons, reduce microgliosis, and shift microglial activation toward a less inflammatory state in preclinical models of neurodegeneration [77]. While these findings suggest that flavonoids may mitigate neuromuscular decline, evidence of synergistic effects from combining flavonoids is lacking, and the oversimplified M1/M2 microglial model does not fully capture the complexity of microglial dynamics. Together, these data suggest that nutrigenetic approaches leveraging bioactive compounds such as kiwifruit and flavonoids hold potential to complement gene-modulating therapies in SMA by targeting neurodegeneration and metabolic dysfunction. The preclinical data are promising, but human clinical trials are needed to validate efficacy, optimize dosing, and assess safety. Future research should focus on identifying genetic and metabolic biomarkers to guide personalized dietary interventions, ultimately improving quality of life and long-term outcomes for SMA patients.

### 5.5. Nutrients and Muscular Dystrophy

In some models of MD, it has been demonstrated that dietary interventions using bioactive compounds, such as polyphenols and omega-3 fatty acids, show promise as adjunct therapies. Polyphenols from green tea extract (GTE) and cocoa exhibit protective effects in the mdx mouse model of DMD. (-)-Epigallocatechin gallate (EGCG), a major GTE component, reduces muscle fiber necrosis, enhances muscle resilience, and delays disease onset [78,79]. Similarly, (-)-epicatechin (EC), the primary flavonoid in cocoa, improves mitochondrial function, mitigates oxidative stress, and reduces skeletal and cardiac muscle fibrosis [80]. EGCG may also modulate epigenetic pathways by inhibiting DNMTs through direct binding or the production of S-adenosyl-L-homocysteine (SAH), potentially upregulating genes involved in muscle repair [81]. However, direct evidence linking DNMT inhibition to muscle regeneration in DMD is lacking. EC upregulates myogenic regulators, such as follistatin and MEF2, in preclinical models, but its relevance to DMD versus aging-related muscle decline requires further investigation [82].

Omega-3 fatty acids, including eicosapentaenoic acid (EPA) and docosahexaenoic acid (DHA), offer anti-inflammatory benefits in DMD. Long-term supplementation in mdx mice reduces nuclear factor kappa B (NF-κB) activation in the diaphragm muscle by inhibiting IκB degradation, thereby lowering inflammation [83]. Omega-3s also modulate mitogen-activated protein kinase (MAPK) pathways, which regulate satellite cell proliferation and muscle repair, though their effects vary by context [84]. Additionally, omega-3 supplementation decreases serum creatine kinase levels and improves limb strength in mdx mice [85]. Resveratrol, another polyphenol, supports muscle homeostasis by stimulating protein synthesis, suggesting broader potential for dietary interventions [86].

Thus, dietary polyphenols (EGCG, EC, resveratrol) and omega-3 fatty acids show promise in preclinical DMD models by reducing inflammation, oxidative stress, and fibrosis while promoting muscle repair. However, their clinical efficacy remains unproven due to the lack of human trials, variable bioavailability, and potential interactions with standard therapies. Future research should prioritize clinical studies to validate these interventions and elucidate their mechanisms, paving the way for personalized dietary strategies to complement existing DMD treatments.

**Table 4:** Nutriepigenetic modulators in neuromuscular disorders (NMDs).

Disorder	Nutrient/Bioactive Compound	Key Findings	Mechanism of Action	Epigenetic/Molecular Effects	References
Amyotrophic Lateral Sclerosis (ALS)	Curcumin	Improves survival in ALS patients and reduces SOD1/TDP-43 aggregation	Inhibits DNMTs and HDACs; chelates metals	Increases H4 acetylation, elevates SOCS1/SOCS3 levels, and decreases pro-apoptotic methylation	[68,69]
	Dietary Restriction	Induces autophagy and removes toxic protein aggregates	Activates AMP-activated protein kinase (AMPK) and mechanistic target of rapamycin (mTOR) pathways	Enhances clearance of misfolded proteins such as SOD1 and TDP-43	[71,74]
Spinal Muscular Atrophy (SMA)	Kiwifruit Extract	Improves motor neuron survival in a *C. elegans* SMA model	Exhibits antioxidant and anti-inflammatory effects	Modulates oxidative stress pathways	[76]
	Green Tea and Cocoa Flavonoids	Stabilize motor neuron synapses and attenuate microgliosis	Balance M1 and M2 microglial phenotypes	Enhance synaptic stability and reduce neuroinflammation	[77]
Muscular Dystrophy (MD)	Green Tea Epigallocatechin Gallate (EGCG)	Reduces muscle necrosis and delays the onset of dystrophic symptoms	Inhibits DNMT activity via S-adenosylhomocysteine (SAH) production	Increases the expression of myogenic genes (Myf5, MEF2) and decreases fibrosis	[79]
	Cocoa (-)-Epicatechin (EC)	Improves mitochondrial function and reduces fibrosis	Increases follistatin levels and decreases myostatin	Promotes satellite cell differentiation	[80,82]
	Omega-3 Fatty Acids (EPA/DHA)	Reduce NF-κB-mediated inflammation and improve muscle strength	Inhibit IκB degradation and modulate mitogen-activated protein kinase (MAPK)	Decrease serum creatine kinase (CK) levels and enhance muscle repair	[83]
	Resveratrol	Stimulates protein synthesis and supports muscle homeostasis	Activates sirtuin 1 (*SIRT1*) and peroxisome proliferator-activated receptor γ coactivator 1-α (*PGC-1α*)	Enhances mitochondrial biogenesis	[86]

## 6. Conclusions

Neuromuscular disorders, such as ALS, SMA, and MD, arise from complex genetic and epigenetic interactions that influence disease progression and therapeutic responses. Our review highlights the importance of genetic modifiers, exercise-induced epigenetic modifications, the regulatory role of dietary bioactive compounds, and nutrient-gene interactions with potential disease-specific benefits. While these factors offer promising avenues for intervention, their clinical application remains underexplored. Future research should prioritize mechanistic studies and translational approaches to refine therapeutic strategies, increase evidence base, and improve patient outcomes. Therefore, increasing evidence is essential to promote their use and bridge the gap between experimental findings and clinical practice.

## Figures and Tables

**Table 2:** Key genetic and epigenetic findings in SMA.

Category	Key Factor	Effect on SMA	Mechanism/Evidence	References
Genetic Basis	*SMN1* deletion	Results in loss of functional SMN protein	This is the primary cause of spinal muscular atrophy (SMA)	[4,43]
Disease Modifier	*SMN2* copy number	Higher copy number is associated with a milder phenotype	*SMN2* compensates for the loss of *SMN1* through alternative splicing	[43]
Epigenetic Regulation	*SMN2* methylation	Hypermethylation leads to reduced expression	Methylation status correlates with disease severity	[44]
Epigenetic Regulation	miRNAs	Potential biomarkers	The most frequently deregulated miRNAs in SMA patients include miR-1-3p, miR-133a-3p, miR-133b, and miR-206	[47]
iPSC Studies	*PAX6*, *HB9*, *CHAT* methylation	Alters motor neuron differentiation	Indicates epigenetic dysregulation in SMA neurons	[46]
